# Effect of *Opuntia humifusa* Supplementation and Acute Exercise on Insulin Sensitivity and Associations with PPAR-γ and PGC-1α Protein Expression in Skeletal Muscle of Rats

**DOI:** 10.3390/ijms14047140

**Published:** 2013-03-28

**Authors:** Junyong Kang, Junghun Lee, Daekeun Kwon, Youngju Song

**Affiliations:** Laboratory of Sports Nutrition, Sunmoon University, Asan 336-708, Korea; E-Mails: garcia60@sunmoon.ac.kr (J.K.); knupeboy@naver.com (J.L.); ksunsu@hanmail.net (D.K.)

**Keywords:** *O. humifusa*, insulin sensitivity, PPAR-γ, PGC-1α, GLUT-4

## Abstract

This study examined whether *Opuntia humifusa* (*O. humifusa*), which is a member of the Cactaceae family, supplementation and acute swimming exercise affect insulin sensitivity and associations with PPAR-γ and PGC-1α protein expression in rats. Thirty-two rats were randomly divided into four groups (HS: high fat diet sedentary group, *n* = 8; HE: high fat diet acute exercise group, *n* = 8; OS: 5% *O. humifusa* supplemented high fat diet sedentary group, *n* = 8; OE: 5% *O. humifusa* supplemented high fat diet acute exercise group, *n* = 8). Rats in the HE and OE swam for 120 min. before being sacrificed. Our results indicated that serum glucose level, fasting insulin level and homeostasis model assessment of insulin resistance (HOMA-IR) in OS were significantly lower compared to those of the HS (*p* < 0.01, *p* < 0.05, *p* < 0.05). In addition, PPAR-γ protein expression in the OS and OE was significantly higher than that of the HS and HE, respectively (*p* < 0.05, *p* < 0.01). PGC-1α and GLUT-4 protein expressions in the OS were significantly higher compared to those of the HS (*p* < 0.05, *p* < 0.05). From these results, *O. humifusa* supplementation might play an important role for improving insulin sensitivity through elevation of PPAR-γ, PGC-1α, and GLUT-4 protein expression in rat skeletal muscle.

## 1. Introduction

Obesity is a serious health problem that increases the risk factors of various metabolic diseases such as type II diabetes (T2D). T2D is characterized by increased blood glucose levels which arise primarily from peripheral resistance to insulin in fat and muscle due to dysfunction of insulin action [1,2], and is also caused by reduction of hepatic insulin sensitivity which can lead to increased output of glucose by increased rate of hepatic glucose production in liver [3]. T2D is the most common form of diabetes which is closely associated with obesity and weight gain [4,5]. For individuals with diabetes, physical activity, dietary control, and drug treatment are an important part of managing the disease.

Thiazolidinediones (TZDs), which are peroxisome proliferator-activated receptor-γ (PPAR-γ) agonists [6,7] that decrease insulin resistance [8], are widely used as a treatment for patients with T2D. Side effects of TZDs, obtained though chemical synthesis, are not fully understood; however, some studies have reported adverse effects from taking TZDs [9,10]. Recently, studies have examined the physiological and pharmacological effects of using therapeutics for T2D that are derived from wild plants as a natural source. In a previous study, Song *et al*., reported that soluble dietary fiber (psyllium) supplementation has a protective effect from the development of insulin resistance through the elevation of skeletal muscle glucose transporter-4 (GLUT-4) protein expression in stroke-prone spontaneously hypertensive rats [11]. Furthermore, long-term supplementation of Korean red ginseng in high fat diet-induced obese rats improved their insulin sensitivity by enhancing muscle GLUT-4 translocation to the plasma membrane through the insulin signaling pathway [12].

Approximately 4000 types of cacti exist and are mostly grown in semi-arid countries around the world, particularly in Central and South America. Of these, *Opuntia humifusa* (*O. humifusa*) is a member of the Cactaceae family that has been cultivated to grow in cold environments below −20 °C [13] and in large quantities in Asan, Chungnam, South Korea. In particular, *O. humifusa* is repleted of not only Mg^2+^, Ca^2+^, and K^+^[14] but also flavonoids, such as quercetin [15] and is widely used as a nutritional supplementation source. According to the previous studies, however, while *Opuntia ficus-indica* (*O. ficus-indica*), another member of Cactaceae family, is well documented in biochemical, biological, and pharmacological studies regarding anti-inflammatory [16], anti-cancer [17], and antioxidant effects [18], few studies have been reported in relation to *O. humifusa*[14,15,19]. In a previous study, Hahm *et al*., reported that oral supplementation of suspended *O. humifusa* in distilled water has an antidiabetic effect due to reduced blood glucose levels in streptozotocin (STZ)-induced diabetic rats by increasing the relative beta cell volume in pancreas [19].

It is well established that moderate intensive exercise improves insulin sensitivity. A previous study reported that acute exercise enhance glucose uptake into mouse skeletal muscle via insulin-dependent and -independent signal transduction mechanisms [20]. In addition, Ruschke *et al*., reported that individuals with T2D who performed long-term endurance exercise had improved insulin sensitivity due to an increase in PPAR-γ and peroxisome proliferator-activated receptor gamma coactivator 1-alpha (PGC-1α) protein expression [21]. However, to our knowledge, there have not been any reports studying the effects of *O. humifusa* supplementation on skeletal muscle protein expression of PPAR-γ and PGC-1α and its relation to insulin sensitivity or obesity as well as the potential synergic effect between *O. humifusa* supplementation and exercise.

Meanwhile, PPAR-γ is the member of nuclear receptor gene family. Although PPAR-γ is more highly expressed in adipose tissue than in muscle, muscle specific-PPAR-γ deletion is susceptible to developing insulin resistance in mice [22]. In addition, PPAR-γ activation plays an important role for the improvement of insulin sensitivity as well as regulation of GLUT-4 gene expression in skeletal muscle [23]. A relationship with PGC-1α and insulin resistance has also been shown, Patti *et al*., reported that reduced PGC-1α expression is related to the development of insulin resistance due to decreased expression of a nuclear respiratory factor (NRF)-dependent gene [24]. Furthermore, over-expression of PGC-1α has been shown to increase the level of insulin-regulated GLUT-4 mRNA and glucose uptake [25].

The aim of this study was to investigate that the effect of *O. humifusa* supplementation and acute swimming exercise on insulin sensitivity and associations with PPAR-γ and PGC-1α protein expression in the skeletal muscle of rats fed a high fat diet.

## 2. Results

### 2.1. Body Weight, Food Efficiency Ratio (FER) and Fat Tissue Weight

As shown in Table 1, the initial and final body weights were not significantly different among groups. Additionally, the FER and fat tissue weights were not significantly different among groups.

### 2.2. Changes in Serum Parameters and HOMA-IR

**Figure 1:**
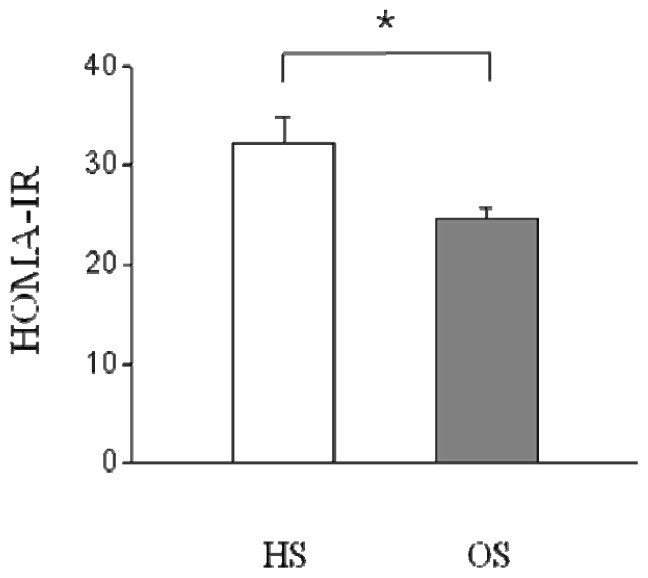
Effect of *O. humifusa* supplementation on HOMA-IR. HS, high fat diet sedentary group; OS, *O. humifusa* supplemented high fat diet sedentary group. *p < 0.05.

As shown in Table 2, serum glucose, insulin and triglyceride (TG) levels in the OS were significantly lower than those of the HS (*p* < 0.05, *p* < 0.01). In addition, Serum insulin, TG, free fatty acid (FFA) and total cholesterol (TC) levels observed in the OE were significantly different compared to those of the OS (*p* < 0.01); additionally serum TG and FFA levels measured in the OE were found to be significantly higher than those of the HE (*p* < 0.05, *p* < 0.01). Serum glucose, insulin, TG and FFA levels insulin levels observed in the OE were significantly different compared to those of the HS (*p* < 0.01). In addition, serum insulin and FFA levels of OS were significantly different than those of HE (*p* < 0.01). HOMA-IR of the OS was significantly decreased relative to that of the HS (*p* < 0.05, Figure 1).

### 2.3. Skeletal Muscle PPAR-γ, PGC-1α, and GLUT-4 Protein Expressions

**Figure 2:**
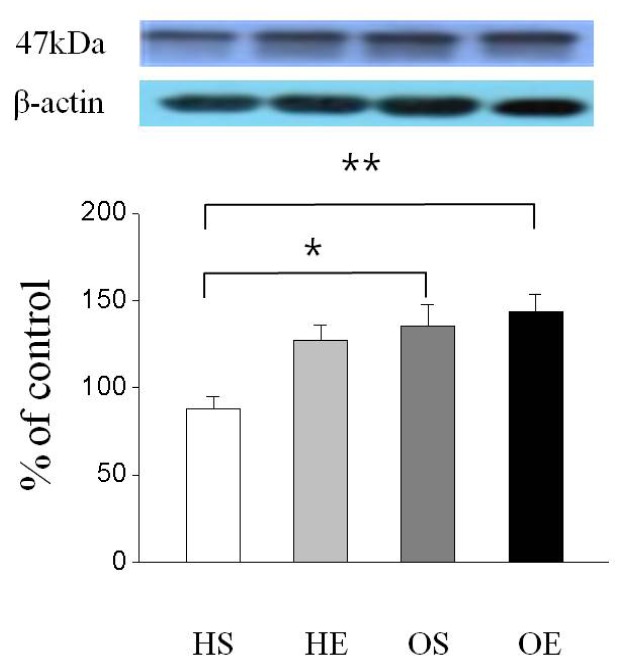
Effect of *O. humifusa* supplementation on GLUT-4 protein expression in skeletal muscle. HS, high fat diet sedentary group; HE, high fat diet acute exercise group; OS, *O. humifusa* supplemented high fat diet sedentary group; OE, *O. humifusa* supplemented high fat diet acute exercise group. *p < 0.05; **p < 0.01.

**Figure 3:**
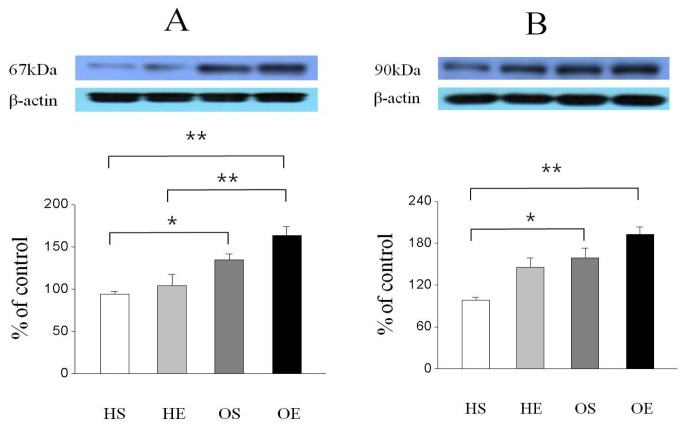
Effect of *O. humifusa* supplementation on PPAR-γ and PGC-1α protein expressions in skeletal muscle. HS, high fat diet sedentary group; HE, high fat diet acute exercise group; OS, *O. humifusa* supplemented high fat diet sedentary group; OE, *O. humifusa* supplemented high fat diet acute exercise group. *p < 0.05; **p < 0.01. (**A**) PPAR-γ; (**B**) PGC-1α.

As shown in Figure 2, GLUT-4 protein expression was significantly higher in the OS than in the HS (*p* < 0.05) and OE was significantly higher than that of the HS (*p* < 0.01). In addition, PPAR-γ protein expression was significantly higher in the OS than in the HS (*p* < 0.05, Figure 3A), and the expression level of PPAR-γ in the OE was significantly higher compared to that of the HE and HS (*p* < 0.01, Figure 3A). Furthermore, PGC-1α protein expression in the OS was significantly higher than in the HS (*p* < 0.05, Figure 3B), and expression of PGC-1α in the OE was significantly higher compared to that of the HS (*p* < 0.01, Figure 3B).

### 2.4. Correlation among Skeletal Muscle PPAR-γ, PGC-1α, and GLUT-4 Protein Expressions

**Figure 4:**
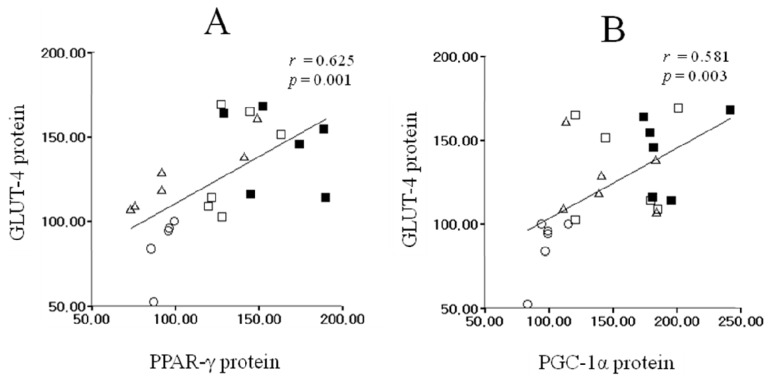
Correlation among PPAR-γ, PGC-1α, and GLUT-4 protein expressions in skeletal muscle. These relationships represented significant positive correlation (Pearson’s correlation coefficient A: *r* = 0.625, *p* = 0.001; B: *r* = 0.581, *p* = 0.003). The equations for the fitted lines are *y* = 0.56*x* + 54.67 (**A**), and *y* = 0.42*x* + 60.98 (**B**). ○: HS, △: HE, □: OS, ■: OE.

As shown in Figure 4A, the increased levels of PPAR-γ and GLUT-4 protein exhibited significant positive correlation (*r* = 0.625, *p* = 0.001). Thus, there was highly significant positive correlation between PGC-1α protein expression and GLUT-4 protein expression (*r* = 0.581, *p* = 0.003, Figure 4B).

## 3. Discussion

To the best our knowledge, this is the first study to investigate the effects of *O. humifusa* supplementation and acute swimming exercise on insulin sensitivity by analyzing skeletal muscle protein expression of rats. In the results, serum glucose level of the OS was significantly lower compared to the level observed in the HS, and the OE tended to have lower value relative to the HE after the eight-week experimental period. In a previous study, it had been suggested that oral supplementation of *O. humifusa* lowered serum glucose level in STZ-induced diabetic Sprague Dawley male rats due to high dietary fiber and other carbohydrate components [19]. Furthermore, some previous reports studying the association between mineral supplementation and risk of T2D found that magnesium-rich foods, such as whole grains, and calcium supplementation can decrease the risk of T2D in humans [26,27]. In addition, serum insulin level and HOMA-IR were showed significantly lower in the OS compared to the HS. Therefore, these results suggest that *O. humifusa* supplementation, which is rich in fiber and minerals [14], could potentially lower glucose levels.

It is well established that the mechanism for elevating glucose uptake is caused by the translocation of GLUT-4 vesicles from intracellular pool to the plasma membrane through insulin binding to its receptor. This leads to tyrosine phosphorylation of insulin receptor substrates (IRS) by tyrosine kinase, and phosphatidylinositol 3-kinase (PI3-kinase) activates 3-phosphoinositide-dependent protein kinase-1 (PDK), which activates Akt. It has been suggested that Mg^2+^ plays an important role in cellular glucose utilization and regulate the insulin action to its receptor as well as insulin signaling mechanisms involved in glucose transport [28]. According to a previous study, lower dietary Mg^2+^ or lower serum Mg^2+^ is associated with an increased risk for T2D [29]. Suarez *et al*., reported that low Mg^2+^ diet leads to impairment of muscle insulin tyrosine activity which may directly affect insulin signaling that is related to insulin resistance [30]. Although exact correlation between PPARs and Mg^2+^ is not clearly understood, an interaction between Mg^2+^ and PPAR-γ receptor has been suggested. A previous study showed that 12 weeks of pioglitazone treatment (30 mg/day), which is a prescription drug of the TZDs class with a high binding affinity for PPAR-γ [31], increased serum Mg^2+^ level by 112% [32,33]. According to a previous study, PPAR-γ protein is expressed at low level in muscle, but PPAR-γ protein is an important role for regulation GLUT-4 gene expression in muscle tissue [23]. However, because elevated states of insulin resistance in skeletal muscle caused impaired function of GLUT-4 or translocation [34], glucose uptake decreased through depression of GLUT-4 function. Previous studies have also suggested a connection between low/insufficient Ca^2+^ intake and the incidence of T2D. It has been shown that elevation in intracellular Ca^2+^ may result in insulin resistance by affecting the phosphorylation of GLUT-4 [35] and affecting insulin-mediated glucose transport and insulin secretion, leading to insulin resistance and T2D [36,37]. However, 8 weeks of oral Ca^2+^ supplementation at 1500 mg per day improved insulin sensitivity by reducing the concentration of intracellular ionic Ca^2+^ in 31 diabetic and hypertensive individuals [38]. Furthermore, a previous study reported that 8 weeks of Ca^2+^ intake improves insulin sensitivity in essential hypertensive patients [39]. In the present study, the mineral composition of the *O. humifusa* was approximately 1200 mg/100 g for Mg^2+^ and approximately 2300 mg/100 g for Ca^2+^. Therefore, supplementation of Mg^2+^ and Ca^2+^ in *O. humifusa* diet groups resulted in approximately 2.2 times the level of Mg^2+^ and 1.3 times the level of Ca^2+^ present as the high fat diet groups 5% *O. humifusa* added to the high fat diet whereas the daily food intakes were similar among four groups. However, the effect of 5% of *O. humifusa* supplementation on insulin sensitivity of human is uncertain. In addition, although we used 5% of *O. humifusa* supplementation, optimal dose of the *O. humifusa* on insulin sensitivity is uncertain because we did not analyze dose response of *O. humifusa* on insulin sensitivity in the present study.

An acute exercise increases skeletal muscle glucose uptake and insulin sensitivity. It has been reported that continuous muscle contraction causes adenosine triphosphate (ATP) degradation and adenosine monophosphate (AMP) accumulation, leading to activation of 5′ AMP-activated protein kinase (AMPK) [40,41]. AMPK activation during the exercise is the major mechanism for enhanced PGC-1α in a skeletal muscle [42] and it has been reported that enhanced PGC-1α expression greatly increased GLUT-4 expression and glucose transport rates in muscle [25]. In the present study, our study showed that muscle GLUT-4 and PGC-1α protein expressions of OE was tend to higher than that of the HE, and PPAR-γ protein expression of OE was significantly higher compared to that of the HE. Previous studies have reported that PPAR-γ, and PGC-1α protein expression is increased with exercise in rodents, [43,44] and human models [45], and is up-regulate by acute exercise [46]. In a previous study, Baar *et al*., reported that a single long-term bout of swimming exercise showed an increase in PGC-1α mRNA in rat skeletal muscle [47]. Furthermore, it has been reported that exhaustive acute exercise can cause elevated of PGC-1α protein expression whereas PPAR-γ was decreased after exhaustive acute exercise in skeletal muscle [48]. However, it is also reported that single bout of endurance exercise for 60 min did not influence PPAR-γ and PGC-1α protein expression [49]. Therefore, we might assume that changes in protein expression are affected by external factors, such as the subject, recruited muscle type during exercise, exercise time and capacity; thus, further studies, considering exercise time and capacity, are needed to better elucidate the relationship between exercise and protein expression.

Our study showed that muscle PPAR-γ protein expression in the OS was significantly higher than that of the HS, and GLUT-4 and PGC-1α protein expression was significantly higher in the OS than that in the HS. According to Loviscach *et al*., [50], PPAR-γ is highly expressed in adipose tissue, whereas low levels of protein are expressed in skeletal muscle. However, although the exact function of PPAR-γ protein in skeletal muscle is still not clearly understood, it has been reported that insulin resistance was increased in a muscle-specific PPAR-γ knock-out mouse model [22]. Furthermore, skeletal muscle comprises a relatively large mass in the body and is an important target tissue for glucose metabolism by insulin [51]. Therefore, we may assume that small changes in PPAR-γ activity might have significant physiological effects because skeletal muscles are the major target tissues of insulin for glucose uptake. In addition, PGC-1α gene is highly expressed in skeletal muscle and insulin stimulation increases muscle PGC-1α expression [52,53]. It has also been suggested that a decline in PGC-1α protein expression due to supplementation of a high fat diet caused an increase in insulin resistance in mouse muscle tissue [54]. Furthermore, as the results of the present study showed, there was a positive correlation observed between PPAR-γ, PGC-1α, and GLUT-4 protein expression which suggesting the potential mechanism that an increase in GLUT-4 protein expression might be attributable to be through PPAR-γ-mediated and PGC-1α-pathway. Although we demonstrated that up-regulation of PPAR-γ, PGC-1α and GLUT-4 protein expression by *O. humifusa* supplementation, the exact mechanism is not fully understood in the present study. Therefore, further studies are also needed to elucidate the effect of *O. humifusa* supplementation on insulin sensitivity such as AMPK, nicotinamide adenine dinucleotide (NAD)-dependent deacetylase (SIRT1) and membrane translocation of GLUT-4. In addition, serum insulin level and HOMA-IR were showed significantly lower in the OS compared to the HS. Therefore, supplementation of *O. humifusa* leads to up-regulation of glucose uptake through the increase in PPAR-γ, PGC-1α and GLUT-4 protein expressions, consequently resulting in improved insulin sensitivity in rat skeletal muscle. However, although freeze-dried raw *O. humifusa* was tested, future studies according to different types of preparation as well as compare to other botanical extracts are also needed to more elucidate the effect of *O. humifusa* supplementation on insulin sensitivity. Finally, although the PGC-1α and GLUT-4 protein expressions of both *O. humifusa* and acute exercise supplementation groups were tend to higher compare to those of the *O. humifusa* supplementation groups, the results indicated that there was no synergic effect between *O. humifusa* supplementation and acute bout of exercise.

## 4. Materials and Methods

### 4.1. Experimental Animals and Exercise Protocol

All experimental protocols were approved by the Animal Study Committee of Sunmoon University. After the acclimatization period during week one, thirty-two 6-week-old male Sprague Dawley rats (Samtaco Bio Korea, Hwaseong, Korea) were randomly divided into four groups; HS: high fat diet sedentary group (*n* = 8), HE: high fat diet acute exercise group (*n* = 8), OS: 5% *O. humifusa* supplemented high fat diet sedentary group (*n* = 8), and OE: 5% *O. humifusa* supplemented high fat diet acute exercise group (*n* = 8) and given the experimental diets for 8 weeks and free access to tap water during the experimental period. Rats were housed in groups of two per cage at a controlled temperature (23 ± 1 °C) and relative humidity (50% ± 5%). The light/dark cycle was automatically controlled (alternating 12-h periods), and lighting was begun at 8:00 pm. The amount of daily food intake was measured daily and body weight was measured weekly. FER was calculated as the total weight gained divided into total food intake for the experimental period. At the end of the experimental period, the sedentary rats were killed by withdrawing blood from the left ventricle under light diethyl ether anesthesia after fasting for 12 h. The exercise groups were acclimated to swimming exercise for 10 min/day for 3 days and 2 days before performing acute swimming exercise, the last acclimation training was finished to wash out any pre-conditioning training effects. On the experiment day, the rats in acute exercise groups swam for 120 min with 2% load/weight in a large plastic tube filled with water (temp.: 35 ± 1 °C; depth: 50 cm; radius: 25 cm) after fasting for 12 h. The rats were then killed in the same way as the sedentary rats after two hours recovery following exercise, and serum was obtained by centrifuging the blood at 700× *g* for 20 min at 4 °C. Both hind limb muscles were dissected and immediately immersed in liquid nitrogen. The serum samples and hind limb muscles were stored at −70 °C until analyzed.

### 4.2. Preparation of Experimental Diet

*O. humifusa*, which was harvested in Asan, Chungnam, was cleaned and blended using a HMF-3150S blender (Hanil Electronics, Seoul, Korea). After blending, the *O. humifusa* was frozen in a freezer at a temperature of −70 °C and then freeze-dried in a freeze dryer (Ilshin Co., Gyeonggi, Korea). After freeze-drying, as shown in Table 3, a general component analysis of *O. humifusa* was performed using the association of official analytical chemists (AOAC) method [55] for the following measurements: moisture using an air-oven method, ash using dry ashing a method, carbohydrates using calculation, crude protein using a kjeldahl method, crude fat using a soxhlet extraction method, and fiber using an enzymatic-chemical method. Mineral component analysis was performed using plasma atomic emission spectrometry (ICP-AES) to determine the composition with respect to Fe^2+^, Ca^2+^, Mg^2+^, K^+^, Na^+^ and P^2+^. As shown in Table 4, the high fat diet was composed of 20% protein, 48% carbohydrate and 20% fat which is modified from a previous study [56] and based on AIN-76G; and the 5% *O. humifusa* diet was made by substituting a portion of carbohydrate, protein, fiber, and fat components of control diet. During the experimental period, the diet was prepared in batch for 3~5 days, and the experimental diets were stored at 4 °C to maintain freshness.

### 4.3. Serum Analysis

Serum glucose, TG, TC, and HDLC levels were analyzed using enzymatic kits (Asan Pharmaceutical Co, Yongin, Korea). Fasting insulin level was measured using a Coat-A-Count RIA Kit (Linco Research, Inc., St. Louis, MO, USA), and insulin resistance was calculated according to homeostasis model assessment of insulin resistance (HOMA-IR) using the following formula: [fasting glucose level (mmol/L) × fasting insulin level (μU/mL)]/22.5. Serum FFA concentration was measured using a Wako NEFA commercial test kit (Wako Chemical, Tokyo, Japan).

### 4.4. Western Blot Analysis

For protein expression analysis, soleus muscle was homogenized on ice with a polytron homogenizer in 20 mmol/L Tris-HCl buffer (pH7.5) containing 5 mmol/L EDTA, 2 mmol/L PMSF, 1:200 protease inhibitor cocktail (Sigma, St. Louis, MO, USA). The protein concentrations were determined using a Bradford reagent from Bio-Rad (USA), with bovine serum albumin as the standard. An aliquot of tissue extract containing 20 μg (PPAR-γ, PGC-1α, GLUT-4) of protein was separated on a 10% SDS-PAGE gel. After electrophoresis, the proteins were transferred to a PVDF membrane (Millipore, Bedford, MA, USA) in a semi-dry blotting apparatus (Bio-Rad, Hercules, CA, USA). After treating with blocking buffer (PBS containing 10% skim milk) for 90 min, the membrane was incubated with primary polyclonal antibodies for 2 h (PPAR-γ; PGC-1α; GLUT-4; Santa Cruz, CA, USA), followed by five 10 min. washes with PBS (5% tween 20). The membrane was then incubated with HRP-conjugated anti-goat IgG or anti-rabbit IgG (Santa Cruz, CA, USA) for 1 h, followed by five 10 min. washes with PBS (5% tween 20). The target proteins were detected using an ECL kit (Amersham Pharmacia Biotech, Piscataway, NJ, USA). The films were photographed and the protein bands of interest were quantified with band analyzer software (Bio-Rad, Hercules, CA, USA).

### 4.5. Statistical Analysis

All data were analyzed using SPSS software (version 15.0 for Windows). The data are expressed as the means ± SE, and values were analyzed by one-way ANOVA followed by Scheffe’s test. Thus, linear relationships between PPAR-γ, PGC-1α and GLUT-4 protein expression were examined using Pearson’s correlation analysis. Significance was defined as α = 0.05.

## 5. Conclusions

The results of the present study suggested that increased PPAR-γ, PGC-1α, and GLUT-4 protein expressions by *O. humifusa* supplementation are responsible for the favorable impact on insulin sensitivity.

## Figures and Tables

**Table 1:** Changes in body weight, food efficiency ratio (FER), and fat tissue weights.

	HS	HE	OS	OE
Initial body weight (g)	254.8 ± 3.66	255.4 ± 5.52	255.3 ± 2.46	254.7 ± 5.45
Final body weight (g)	445.9 ± 13.21	454.7 ± 12.33	448.3 ± 9.70	444.9 ± 6.99
Food efficiency ratio	0.23 ± 0.010	0.24 ± 0.010	0.23 ± 0.009	0.23 ± 0.011
Abdominal fat tissue (g)	9.1 ± 0.80	8.2 ± 0.62	9.4 ± 0.66	8.7 ± 0.66
Epididymal fat tissue (g)	8.5 ± 0.75	8.0 ± 0.83	8.6 ± 0.61	8.4 ± 0.43

Values are the means ± SE. HS, high fat diet sedentary group; HE, high fat diet acute exercise group; OS, *O. humifusa* supplemented high fat diet sedentary group; OE, *O. humifusa* supplemented high fat diet acute exercise group.

**Table 2:** Changes in Blood Components.

	HS	HE	OS	OE
Glucose (mg/dL)	157.8 ± 5.05	122.1 ± 2.63 #	119.1 ± 2.16 **	107.2 ± 5.99 §§
Insulin (ng/mL)	3.7 ± 0.26	0.8 ± 0.12 ##	2.5 ± 0.37 *,§§	0.4 ± 0.05 ##,§§
TG (mg/dL)	52.4 ± 4.66	46.2 ± 3.42	30.9 ± 2.66 *	77.0 ± 5.88 **,##,§§
FFA (mEq/L)	319.4 ± 8.55	438.1 ± 13.81 #	328.6 ± 21.80 §§	560.8 ± 29.48 **,##,§§
TC (mg/dL)	77.1 ± 2.06	82.1 ± 5.80	68.5 ± 3.12	87.9 ± 4.63 #
HDLC (mg/dL)	23.4 ± 0.39	23.9 ± 0.70	23.9 ± 1.29	22.8 ± 0.94

Values are the means ± SE. HS, high fat diet sedentary group; HE, high fat diet acute exercise group; OS, *O. humifusa* supplemented high fat diet sedentary group; OE, *O. humifusa* supplemented high fat diet acute exercise group; HDLC, high density lipoprotein cholesterol;

* *p* < 0.05,

** *p* < 0.01 same condition with different diet;

# *p* < 0.05,

## *p* < 0.01 same diet with different condition;

§§ *p* < 0.01 different condition with different diet.

**Table 3:** Compositions of freeze-dried *O. humifusa*.

Ingredients	Contents
moisture (% *w*/*w*)	2.9
ash (% *w*/*w*)	13.8
carbohydrate (g/100 g)	46.6
crude protein (g/100 g)	4.9
crude fat (g/100 g)	3.1
fiber (g/100 g)	28.9
Fe^2+^ (mg/g)	5.8
Ca^2+^ (mg/100 g)	2931.3
Mg^2+^ (mg/100 g)	1227.9
K^+^ (mg/100 g)	2155.5
Na^+^ (mg/100 g)	30.9
P^2+^ (mg/100 g)	653.2

**Table 4:** Compositions of experimental diet (g/kg diet).

Ingredients	High fat diet	5% *O. humifusa* added diet
Casein	200	197.6
Starch	111	87.8
Sucrose	370	370
Lard	170	170
Corn oil	30	28.4
Cellulose	50	35.6
Vitamin mix.	12	12
Mineral mix.	42	42
Cholesterol	10	10
d,l-methionine	3	3
Choline barbiturate	2	2
Tert-butylhydroquinone	0.04	0.04
*O. humifusa*	·	50
